# Supplementary material for: Dietary Patterns and Major Depression: Results from 15,262 Participants (International ALIMENTAL Study)
Source: Nutrients. 2025 May 4;17(9):1583. doi: 10.3390/nu17091583 (PMC12073559; doi:10.3390/nu17091583)
Supplement: Supplementary file 1 [file nutrients-17-01583-s001.zip › nutrients-3607421-supplementary.pdf]

Supplementary material S1. Diet questionnaire.

1. Carbohydrates: fill in the table below according to what you have consumed in the last month:

	**Less than once a week**	**1 to 2 times a week**	**3 to 4 times a week**	**5 to 6 times a week**	**1 time per day**	**2 to 3 times a day**	**4 times a day and more**
**Fast or hidden carbohydrates**							
White bread, rusks, cereals *(except oatmeal)*	☐	☐	☐	☐	☐	☐	☐
Potato *(baked, boiled)*, fresh or white pasta *(= not whole meal or semi-complete)*, white rice	☐	☐	☐	☐	☐	☐	☐
Jam, spread (Nutella® type) *(teaspoon)*	☐	☐	☐	☐	☐	☐	☐
Sweetened soda (including Redbull® and related)	☐	☐	☐	☐	☐	☐	☐
Light or zero soda	☐	☐	☐	☐	☐	☐	☐
Fruit juice (pressed, industrial)	☐	☐	☐	☐	☐	☐	☐
Cream desserts, sweet desserts, ice cream	☐	☐	☐	☐	☐	☐	☐
Yogurt with fruit or flavouring or cottage cheese with fruit or flavouring	☐	☐	☐	☐	☐	☐	☐

2. Protein: fill in the table below according to what you have consumed in the last month:

	**Less than once a week**	**1 to 2 times a week**	**3 to 4 times a week**	**5 to 6 times a week**	**1 time per day**	**2 to 3 times a day**	**4 times a day and more**
**Protein**							
Red meat *(beef, veal, pork, lamb, mutton, horse, goat, duck)*	☐	☐	☐	☐	☐	☐	☐
Unprocessed white meat *(chicken, turkey, rabbit)*	☐	☐	☐	☐	☐	☐	☐
Legumes *(lentilss, chickpeas, white beans, red beans)*	☐	☐	☐	☐	☐	☐	☐
Egg white	☐	☐	☐	☐	☐	☐	☐
Egg yolk (*check the same box as the previous item if all eggs are consumed whole)*	☐	☐	☐	☐	☐	☐	☐
Glass of milk (whole, skimmed or semi-skimmed)	☐	☐	☐	☐	☐	☐	☐
Plain yogurt or plain cottage cheese	☐	☐	☐	☐	☐	☐	☐
Cheese *(portion)*	☐	☐	☐	☐	☐	☐	☐

Protein supplements (powders, tablets, protein bars or other forms)	☐	☐	☐	☐	☐	☐	☐

3. Fibres: fill in the table below according to what you have consumed in the last month:

	**Less than once a week**	**1 to 2 times a week**	**3 to 4 times a week**	**5 to 6 times a week**	**1 time per day**	**2 to 3 times a day**	**4 times a day and more**
**Fibres**							
Green salad or endives	☐	☐	☐	☐	☐	☐	☐
Green vegetables *(green beans, broccoli, asparagus* etc.*)*	☐	☐	☐	☐	☐	☐	☐
Quinoa, bulgur, semolina	☐	☐	☐	☐	☐	☐	☐
Wholemeal or semi-complete rice	☐	☐	☐	☐	☐	☐	☐
Wholemeal or semi-wholemeal pasta	☐	☐	☐	☐	☐	☐	☐
Oat flakes	☐	☐	☐	☐	☐	☐	☐
Fruit	☐	☐	☐	☐	☐	☐	☐
Wholemeal bread	☐	☐	☐	☐	☐	☐	☐

4. Omega 3 and saturated fats: fill in the table below according to what you have consumed in the last month:

	**Less than once a week**	**1 to 2 times a week**	**3 to 4 times a week**	**5 to 6 times a week**	**1 time per day**	**2 to 3 times a day**	**4 times a day and more**
**Omega-3 and saturated fats**							
Tablespoon of olive, rapeseed or soybean oil	☐	☐	☐	☐	☐	☐	☐
Oily fish eaten raw *(sardines, salmon, mackerel, tuna, only fresh and canned, not frozen)*	☐	☐	☐	☐	☐	☐	☐
Other fish and seafood *(not listed above) including canned and frozen*	☐	☐	☐	☐	☐	☐	☐
Nuts, almonds or hazelnuts *(handful of about 6 units)*	☐	☐	☐	☐	☐	☐	☐
Chia seeds	☐	☐	☐	☐	☐	☐	☐
Omega 3 (in capsules or syrup)	☐	☐	☐	☐	☐	☐	☐
Butter (including cooking)	☐	☐	☐	☐	☐	☐	☐

5. Processed food: fill in the table below according to what you have eaten in the last month:

	Less than once a week	1 to 2 times a week	3 to 4 times a week	5 to 6 times a week	1 time per day	2 to 3 times a day	4 times a day and more
**Processed food**							
"Junk food" *(McDonald's, KFC, Burger King, snacks* etc.*)*	☐	☐	☐	☐	☐	☐	☐
Industrially processed meat (salting, curing, fermentation, smoked) (e.g., *ham, slices of chicken fillet, turkey, hot dogs (frankfurters), ham, sausages, corned beef, beef jerky, canned meats)*	☐	☐	☐	☐	☐	☐	☐
Pre-cooked dishes *(canned, tray, frozen)*	☐	☐	☐	☐	☐	☐	☐
Fried foods *(frozen and deep-fried chips, breaded fish, nuggets)*	☐	☐	☐	☐	☐	☐	☐
Grilled appetizers *(peanuts, almonds, roasted pistachios)*	☐	☐	☐	☐	☐	☐	☐
Pastry, cakes, sweet biscuits	☐	☐	☐	☐	☐	☐	☐
Chips, crackers	☐	☐	☐	☐	☐	☐	☐
Margarine (including baking)	☐	☐	☐	☐	☐	☐	☐
Uncooked canned food (e.g., *corn, white beans,* etc.*)* *excluding meat and fish*	☐	☐	☐	☐	☐	☐	☐
Uncooked frozen foods (e.g., *peas, green beans,* etc.*)* *excluding meat and fish*	☐	☐	☐	☐	☐	☐	☐

6. Drinks: fill in the table below according to what you have consumed in the last month (count one glass/cup for 'once'):

	Less than once a week	1 to 2 times a week	3 to 4 times a week	5 to 6 times a week	1 time per day	2 to 3 times a day	4 times a day and more
**In general**							
Coffee *(cup)*	☐	☐	☐	☐	☐	☐	☐
Tea *(cup)*	☐	☐	☐	☐	☐	☐	☐
Decaffeinated coffee *(cup)*	☐	☐	☐	☐	☐	☐	☐
Red wine *(glass)*	☐	☐	☐	☐	☐	☐	☐
White or rose wine *(glass)*	☐	☐	☐	☐	☐	☐	☐
Beer *(25cl)*	☐	☐	☐	☐	☐	☐	☐
Hard liquor *(1 glass or 3cl)*	☐	☐	☐	☐	☐	☐	☐

Supplementary Table S1. Distribution of participants by country

Overall, N = 15,262

Country	n (%)
Afghanistan	1 (<0.1%)
South Africa	1 (<0.1%)
Algeria	1 (<0.1%)
Germany	96 (0.6%)
Andorra	1 (<0.1%)
Australia	1 (<0.1%)
Austria	1 (<0.1%)
Belgium	43 (0.3%)
Benin	2 (<0.1%)
Burkina Faso	1 (<0.1%)
Cameroon	2 (<0.1%)
Canada	510 (3.3%)
Colombia	1 (<0.1%)
Congo (Democratic Republic of the)	2 (<0.1%)
Côte d'Ivoire	1 (<0.1%)
Spain	5 (<0.1%)
United States of America	4 (<0.1%)
France	14,326 (94%)
Greece	1 (<0.1%)
Guadeloupe	3 (<0.1%)
French Guiana	82 (0.5%)
Israel	2 (<0.1%)
Italy	6 (<0.1%)
Japan	1 (<0.1%)
Jordan	1 (<0.1%)
Liechtenstein	1 (<0.1%)
Lithuania	1 (<0.1%)
Madagascar	1 (<0.1%)
Morocco	2 (<0.1%)
Martinique	11 (<0.1%)
Mayotte	43 (0.3%)
Mexico	1 (<0.1%)
Monaco	1 (<0.1%)
Nicaragua	1 (<0.1%)
New Caledonia	2 (<0.1%)
Netherlands	3 (<0.1%)
French Polynesia	7 (<0.1%)
Réunion	61 (0.4%)
Romania	3 (<0.1%)
United Kingdom of Great Britain and Northern Ireland	3 (<0.1%)
Senegal	1 (<0.1%)
Singapore	1 (<0.1%)

Sweden	1 (<0.1%)
Switzerland	20 (0.1%)
Taiwan (Province of China)	1 (<0.1%)
Vietnam	2 (<0.1%)

Supplementary Table S2. Univariable analyses of the associations between 13 dietary pattern factors and depression in 15,262 participants without chronic physical illness and without psychotropic treatment.

F: Factor calculated in the Principal Component Analysis; depression defined by a Center for Epidemiologic Studies Depression Scale (CES-D) score ≥20. In red, significant associations between dietary patterns and an increased risk of depression (OR > 1, p < 0.05); in green, significant associations between dietary patterns and a decreased risk of depression (OR < 1, p < 0.05).

	Whole sample (n=15,262)															
	Women (n=13107; 85.9%)												Men (n=2155; 14.1%)			
	18-34y				35-54y				≥ 55y				18-34y			
	n = 7912				n= 4273				n= 922				n= 1187			
	n(CES D ≥ 20) = 3201 (40%)				n(CES D ≥ 20) = 1050 (25%)				n(CES D ≥ 20) = 179 (19%)				n(CES D ≥ 20) = 321 (27[illegible]			
	OR	(95% IC)		p	OR	(95% IC)		p	OR	(95% IC)		p	OR	(95% IC)		p
**Dietary pattern factors derived from principal component analysis**																
F1 Healthy diet	0.76	0.73;	0.80	**<0.001**	0.78	0.72;	0.84	**<0.001**	0.79	0.66;	0.95	**0.011**	0.82	0.71;	0.96	**0.011**
F2 Ultra-processed foods	1.25	1.20;	1.31	**<0.001**	1.30	1.20;	1.40	**<0.001**	1.39	1.13;	1.71	**0.002**	1.27	1.14;	1.42	**<0.00[illegible]**
F3 Starchy foods	1.03	0.99;	1.08	0.160	1.05	0.98;	1.13	0.180	1.01	0.84;	1.21	0.890	1.03	0.91;	1.16	0.670
F4 Alcohol and coffee	0.92	0.87;	0.97	**0.003**	1.07	0.99;	1.15	0.076	1.10	0.93;	1.29	0.270	0.97	0.87;	1.07	0.510
F5 Eggs	0.98	0.93;	1.02	0.300	1.09	1.01;	1.17	**0.024**	1.05	0.88;	1.26	0.590	1.02	0.91;	1.14	0.780
F6 Meat	1.02	0.98;	1.07	0.280	0.93	0.86;	1.00	0.060	0.86	0.71;	1.04	0.120	1.10	0.98;	1.24	0.120
F7 High glycemic index foods and processed fat	0.92	0.88;	0.97	**<0.001**	1.00	0.93;	1.07	0.970	1.06	0.91;	1.22	0.480	0.82	0.71;	0.94	**0.005**
F8 Supplements (PUFAs and proteins), chia seeds and oat flakes	1.05	1.00;	1.10	0.069	1.11	1.04;	1.19	**0.003**	1.13	0.97;	1.30	0.110	0.99	0.88;	1.10	0.840
F9 Dairy foods and fruit juice	0.92	0.88;	0.96	**<0.001**	0.94	0.87;	1.01	0.077	0.94	0.79;	1.12	0.500	0.86	0.76;	0.98	**0.023**
F10 Canned and frozen foods	1.06	1.02;	1.11	**0.008**	1.02	0.95;	1.10	0.550	1.06	0.89;	1.25	0.510	1.12	0.99;	1.28	0.076
F11 Fish (fatty and lean)	0.95	0.91;	0.99	**0.041**	0.93	0.86;	0.99	**0.038**	1.13	0.97;	1.33	0.120	0.88	0.77;	1.00	0.050
F12 Sugary or sweetened sodas	1.10	1.06;	1.15	**<0.001**	1.03	0.95;	1.11	0.480	0.91	0.74;	1.11	0.360	1.18	1.05;	1.33	**0.006**
F13 Decaffeinated coffee	1.03	0.98;	1.09	0.220	1.06	1.00;	1.13	0.072	0.98	0.86;	1.11	0.760	1.01	0.86;	1.18	0.910
**Confounding factors**																
Obesity	1.41	1.21;	1.66	**<0.001**	1.42	1.17;	1.71	**<0.001**	1.06	0.59;	1.81	0.840	1.68	0.97;	2.85	0.062
Current daily smoking	1.48	1.32;	1.67	**<0.001**	1.37	1.16;	1.63	**<0.001**	1.37	0.87;	2.09	0.170	1.61	1.17;	2.20	**0.003**
Education level (Academic level)	0.58	0.53;	0.63	**<0.001**	0.72	0.61;	0.84	**<0.001**	0.96	0.64;	1.49	0.870	0.66	0.51;	0.85	**0.002**
Nutrition knowledge	0.67	0.61;	0.74	**<0.001**	0.56	0.46;	0.70	**<0.001**	0.79	0.42;	1.61	0.500	0.58	0.42;	0.79	**<0.00[illegible]**
Partner living at home	0.52	0.47;	0.57	**<0.001**	0.65	0.56;	0.76	**<0.001**	0.55	0.40;	0.77	**<0.001**	0.43	0.32;	0.58	**<0.00[illegible]**

Children at home	0.47	0.41;	0.55	**<0.001**	0.81	0.69;	0.97	**0.019**	1.34	0.94;	1.88	0.100	0.39	0.22;	0.66	**<0.00**[illegible]
Unemployment	1.43	1.28;	1.60	**<0.001**	1.67	1.36;	2.04	**<0.001**	2.62	1.45;	4.64	**0.002**	2.21	1.60;	3.05	**<0.00**[illegible]
Phototype 1 and 2 vs. Other phototypes	1.25	1.13;	1.38	**<0.001**	1.11	0.95;	1.30	0.200	1.26	0.85;	1.85	0.250	1.42	1.04;	1.94	**0.02**[illegible]
Physically inactive	1.12	0.98;	1.28	0.085	1.44	1.21;	1.70	**<0.001**	2.11	1.33;	3.28	**0.002**	1.13	0.72;	1.74	0.59[illegible]
